# Traumatic Chylothorax Following Pulmonary Segmentectomy: A Case Report and Review of Postoperative Investigation and Management

**DOI:** 10.7759/cureus.48213

**Published:** 2023-11-03

**Authors:** Shaylor Klein, Erin Hart

**Affiliations:** 1 Internal Medicine and Emergency Medicine, Jefferson Health Northeast, Philadelphia, USA; 2 General Surgery, Philadelphia College of Osteopathic Medicine, Philadelphia, USA

**Keywords:** postoperative complication, chyle, lung resection, segmentectomy, chylothorax, case report

## Abstract

The incidence of iatrogenic traumatic chylothorax is on the rise secondary to the preferred use of minimally invasive thoracic surgery over thoracotomy. Most reported causes of chylothorax occur following pneumonectomy or lobectomy. There have been no reported cases of traumatic chylothorax following segmentectomy according to our literature review. Complications following lung resection typically include pneumonia, atelectasis, or prolonged air leak. Here, we present a rare case of postoperative chylothorax following minimally invasive segmentectomy to diagnose an enlarging singular pulmonary nodule. This condition was diagnosed with fluid analysis after CT imaging revealed a postoperative unilateral pleural effusion. Interestingly, the patient had a loculated pleural effusion that mimicked a pericardial effusion and empyema. Our patient was managed conservatively with a low-fat diet and short-term pleural drainage without the need for repeat surgical intervention. The importance of imaging interpretation following lung resection along with a working differential diagnosis, appropriate examination, and testing can assist with the diagnosis of this known, but rare, postoperative complication.

## Introduction

Traumatic chylothorax, mainly from postoperative injury (iatrogenic), is becoming one of the leading etiologies, superseding spontaneous and idiopathic causes. Disruption of the thoracic duct during surgical intervention, such as pulmonary resection, causes accumulation of chyle within the pleural cavity. Chyle is a complex fluid formed by ingested triglycerides that are broken down into fatty acids in the intestine, forming chylomicrons. Chylomicrons combine with lymphocytes and are transferred by the lymphatic system into the thoracic duct, ultimately draining into the venous circulation. There is an estimated 3% to 7% risk of chylothorax following pulmonary resection and lymph node dissection. Most cases of postoperative chylothorax present within 10 days after the procedure; 80% are unilateral and more often are right-sided. When suspected, the diagnostic test of choice is thoracentesis with fluid sampling. The fluid classically reveals a milky color. Fluid analysis shows greater than 80% lymphocytes and a pH of 7.4-7.8. Confirmation is made with a pleural triglyceride level greater than 110 mg/dL and total serum cholesterol less than 200 mg. Management includes dietary modification, thoracentesis, octreotide, pleurodesis (chemical/mechanical obliteration of the pleural space), and/or ligation or embolization of the thoracic duct [1].

## Case presentation

Our case is regarding a 68-year-old male with a medical history of chronic obstructive pulmonary disease (COPD), obstructive sleep apnea, and former tobacco use, who, during radiological surveillance, demonstrated an increase in the size of a pulmonary nodule from 9 mm to 12 mm on interval CT imaging. The patient elected to undergo surgical resection for further diagnosis, given concern for malignancy. Robotic-assisted, minimally invasive, right lower lobe segmentectomy (with removal of the superior segment) and lymph node dissection were performed. Four unilateral lymph nodes were excised. The final pathology revealed a benign finding of desquamative interstitial pneumonitis. Postoperatively, a chest tube was placed and removed the next day, and the patient was discharged home.

**Figure 1:**
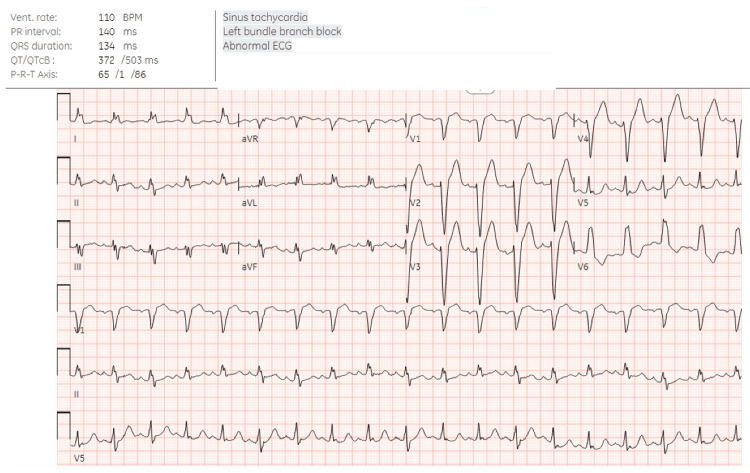
Electrocardiogram obtained in the emergency department revealing sinus tachycardia and complete left bundle branch block.

**Figure 2:**
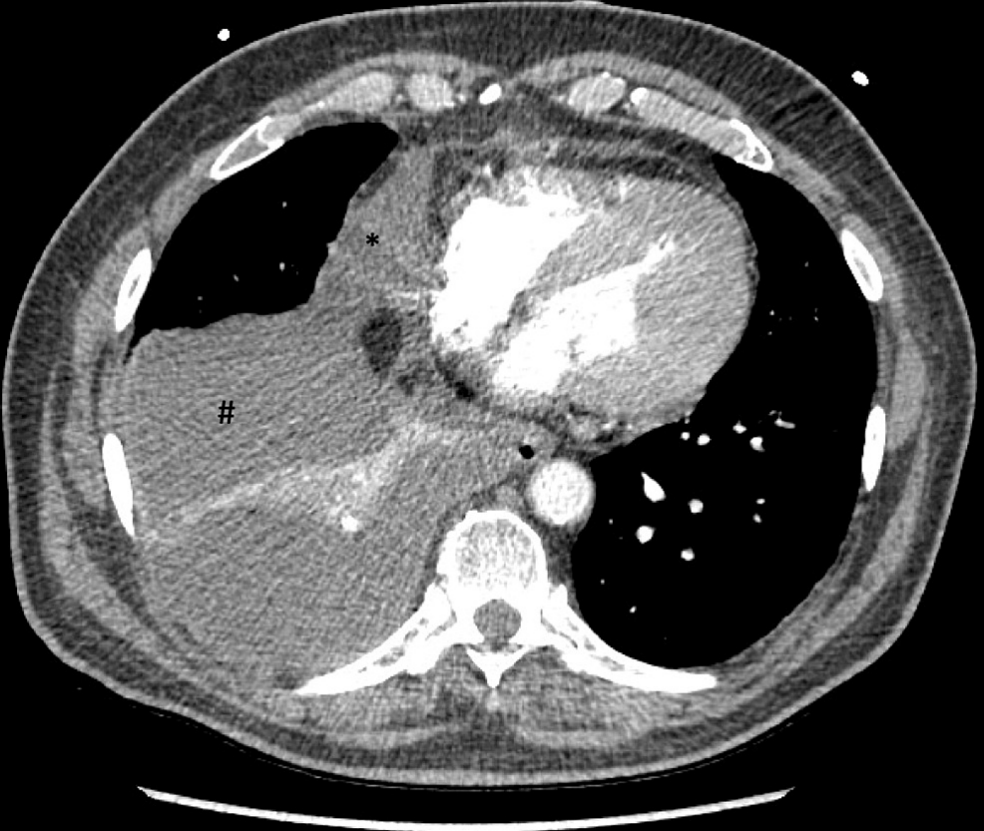
Transverse view of CT angiogram obtained on admission revealing a significant right pleural effusion (#) and right pericardial effusion (*).

**Figure 3:**
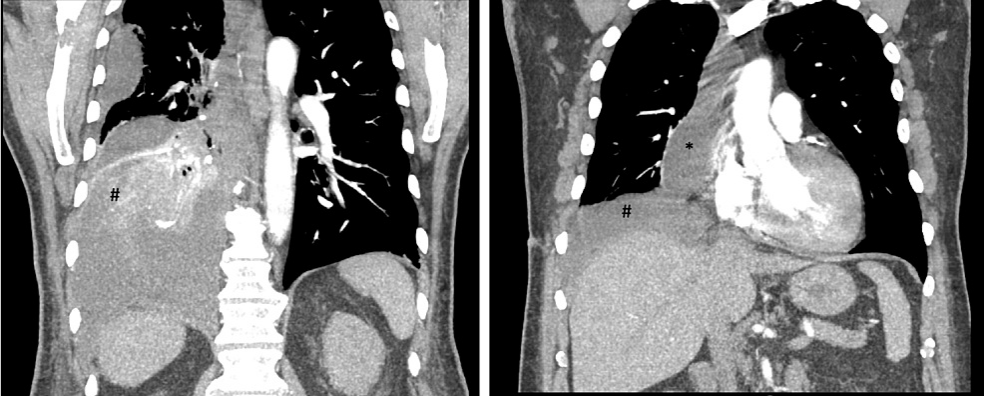
Coronal view of CT angiogram obtained on admission revealing a significant right pleural effusion with loculations (#) and right pericardial effusion (*) measuring up to 4.1 cm.

He subsequently presented to the emergency department on postoperative day (POD) 11 with progressive dyspnea and right chest wall pain. Upon arrival, he was in moderate respiratory distress with an oxygen saturation of 82% on room air, heart rate of 124 beats per minute, blood pressure of 204/80 mmHg, respiratory rate of 40 breaths per minute, and temperature of 97.4°F. He was placed on 4 L of oxygen supplementation via a nasal cannula with improvement in heart rate, oxygen saturation, and tachypnea. A physical examination revealed a well-healing incision to the right mid-axillary chest wall and diminished breath sounds to the right lower lobe. There was no tracheal deviation or jugular venous distention. Labs were significant for leukocytosis of 15,800 white blood cells per liter (B/L) with neutrophilic predominance (83%) and mild anemia with a hemoglobin of 11.2 g/dL. An electrocardiogram revealed a sinus tachycardia and a chronic left bundle branch block but was otherwise without abnormality (Figure 1). Chest X-ray revealed a new right basilar pleural-parenchymal reaction most consistent with effusion and associated atelectasis or pneumonia. A chest CT angiogram revealed a right-sided pleural effusion without tracheal deviation and a large pericardial effusion (Figures 2, 3). He was admitted for acute hypoxic respiratory failure secondary to right-sided pleural effusion.

During his admission, a transthoracic echocardiogram was performed which did not show any significant pericardial effusion. It was thought that this was a loculated pleural effusion overlying the heart (Video 1). On hospital day one, a right-sided 12 French thoracostomy tube was placed by interventional radiology with an immediate output of 400 mL of cloudy yellow fluid. He was started on broad-spectrum antibiotics for presumptive empyema given signs of fluid loculation in the lung. Pleural fluid analysis revealed positive Light’s Criteria with pleural lactate dehydrogenase of 608 IU/L, plasma LDH of 192 IU/L, pleural protein of 4.2 g/dL, and serum protein of 5.5 g/dL. Cell count demonstrated 1,831 white blood cells with a differential of 78% neutrophils, 18% lymphocytes, and 6,000 red blood cells. The pleural fluid triglyceride level was 693 mg/dL, revealing a surprising diagnosis of chylothorax. His total serum cholesterol level was 160 mg/dL. Antibiotics were discontinued at this time and the patient was placed on a low-fat diet. The chest tube was removed on hospital day seven and he was discharged home the following day.

**Video 1:** Four-chamber view during transthoracic echocardiography obtained during admission revealing no pericardial effusion.

## Discussion

Complications after lung resection with video-assisted thoracoscopic surgery appear similar when comparing different types of resections, such as lobectomy or segmentectomy, 38% vs. 33.3%, respectively. Complications, in descending order of likelihood, include, but are not limited to, bronchopneumonia, prolonged air leak, atrial fibrillation, pneumothorax, hemothorax, acute respiratory distress syndrome, atelectasis, acute pulmonary edema, subcutaneous emphysema, chylothorax, pulmonary embolism, bronchopulmonary fistula, and empyema [2]. Another study by Jones et al. revealed segmentectomy complications, in descending order of likelihood, include, but are not limited to, atelectasis, pneumonia, prolonged air leak, and supraventricular arrhythmias [3]. Risk factors for these complications included advanced age (70-79 years old), underweight patients, American College of Anesthesiologists (ASA) class greater than two, increased smoking pack years, diagnosis of COPD, decreased forced expiratory volume in one second (FEV1) and low diffusing capacity of carbon monoxide (DLCO) [2]. Of these complications, chylothorax is rarely encountered, somewhere in the range of 0.25-3% [4]. Specifically, among complications in 690 patients who received lobectomy or segmentectomy, only one patient had a chylothorax which occurred in the lobectomy group [2]. The risk of chylothorax was higher in those who had a lobectomy, robotic resection, or if surgery was performed on the right side [5]. In the available literature, we were unable to find any reports of chylothorax following pulmonary segmentectomy. As such, we report a rare presentation of a patient with postoperative chylothorax following a right lower lobe segmentectomy.

Our patient met the risk factors of ASA class greater than two, 50-100 tobacco pack-year history, COPD with pre-procedure FEV1 of 1.94 L, DLCO of 2.19 mL/minute/mmHg/L, and right-sided surgery. Patients with chylothorax usually present within 10 days of symptoms [1]. Our patient presented on POD 11 but had experienced symptoms for one week before presentation. His course was complicated by the appearance of pleural effusion. His unilateral pleural effusion had multiple loculations that made it appear as though he had an associated large pericardial effusion. There was initial concern that the pericardial effusion would have been a postoperative complication related to either a fistula or hemopericardium. The loculations also gave an appearance of an empyema, triggering the use of broad-spectrum antibiotics and lowering chylothorax as a diagnosis further down on the differential.

**Figure 4:**
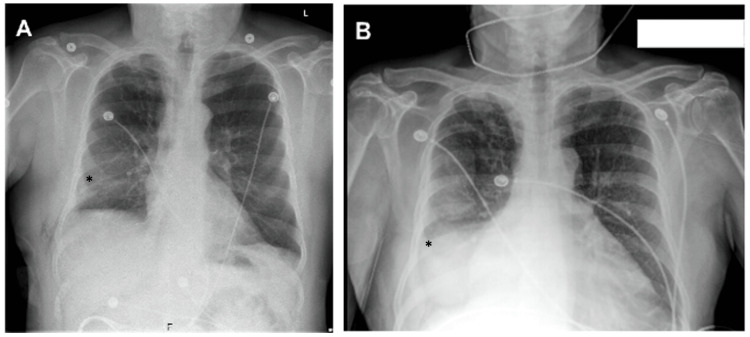
(A) Chest X-ray obtained postoperatively following segmentectomy and chest tube removal revealing normal right-sided subsegmental atelectasis (*). (B) Chest X-ray obtained on return to the emergency department revealing a new right pleural-parenchymal reaction (*) concerning for infection and/or pleural effusion.

It is important to be mindful of physiologic versus pathologic changes when it comes to interpreting studies performed after a patient undergoes lung resection to guide further diagnostic studies and management. When looking at chest X-rays postoperatively, atelectasis is common and can be located anywhere in the chest. Following lung resection, chest X-ray parenchymal opacification is seen commonly with atelectasis and the identification of a pleural effusion can be difficult [6]. Atelectasis can be differentiated from an effusion by mediastinal shift. Those with atelectasis will have a mediastinal shift toward the pathology because of volume loss. A pleural effusion or chylothorax will have a mediastinal shift away from the effusion because it is a space-occupying fluid. For those with a lobectomy/segmentectomy, it is common to experience an ipsilateral elevated diaphragm, a mediastinal shift toward the affected side, and a narrowing of the intercostals. This is more commonly seen in surgeries that remove the lower lobes [6]. Our patient had a right basilar pleural effusion without associated atelectasis/pneumonia on chest X-ray (Figure 4). His CT scan revealed a right-sided pleural effusion without tracheal deviation.

Evaluation of chylothorax involves multiple steps before a definitive diagnosis. With most respiratory complaints, a chest X-ray is primarily obtained. The first finding is typically a unilateral pleural effusion. To further evaluate the lung following a recent thoracic surgery, a CT scan of the chest is obtained. A chylothorax is often described as a “low-attenuation tubular area in the posterior mediastinum” [1]. Along with imaging, laboratory workup is initiated with pleural and serum testing. Thoracentesis is the diagnostic test of choice with a pleural triglyceride level of at least 110 mg/dL and total serum cholesterol less than 200 mg/dL confirming the diagnosis [1], as was the case with our patient.

Treatment of chylothorax can be conservative or interventional. For conservative treatment, patients are managed with low-fat diets. This is related to the composition of chyle and decreasing lymphatic drainage into the thorax [1]. Interventional treatments include initially short-term thoracostomy or interval thoracentesis. If conservative therapy fails, more invasive treatments are explored, such as pleurodesis, thoracic duct ligation, or embolization and disruption. Of note, there is increasing research into using somatostatin/octreotide for chylothorax. The mechanism of action is not entirely understood but may be related to decreasing gastric lymphatic flow, decreasing intestinal fat absorption including triglyceride concentration, and increasing splanchnic blood flow. The lymphatic system may have somatostatin receptor analogs that respond with constriction, further limiting lymphatic flow. The use of this medication, however, appears to have limited research only in pediatrics [7].

## Conclusions

As minimally invasive interventional thoracic therapy expands, postoperative complications should still be considered and not minimalized. Our case report highlights the rarity of a traumatic chylothorax following pulmonary segmentectomy. Maintaining a broad differential to include anything from pneumothorax to empyema to atrial fibrillation and chylothorax is required to best serve our postoperative patient population. Taking steps to correctly identify this disease with imaging and appropriate interpretation, fluid sampling, and laboratory findings is key. Being flexible in diagnostic acumen allows for proper diagnosis and patient safety. Specialist consultation helps determine conservative versus interventional needs. Our patient recovered well with conservative and short-term interventional management. We hope this case presentation was a brief and concise review of postoperative chylothorax after lung resection.

## References

[1] Rudrappa M, Paul M (2023). Chylothorax. https://www.ncbi.nlm.nih.gov/books/NBK459206/.

[2] Bédat B, Abdelnour-Berchtold E, Perneger T (2019). Comparison of postoperative complications between segmentectomy and lobectomy by video-assisted thoracic surgery: a multicenter study. J Cardiothorac Surg.

[3] Jones DR, Stiles BM, Denlinger CE, Antippa P, Daniel TM (2003). Pulmonary segmentectomy: results and complications. Ann Thorac Surg.

[4] Yasuura Y, Konno H, Hayakawa T (2022). Chylothorax after pulmonary resection and lymph node dissection for primary lung cancer; retrospective observational study. J Cardiothorac Surg.

[5] Bryant AS, Minnich DJ, Wei B, Cerfolio RJ (2014). The incidence and management of postoperative chylothorax after pulmonary resection and thoracic mediastinal lymph node dissection. Ann Thorac Surg.

[6] Bommart S, Berthet JP, Durand G, Ghaye B, Pujol JL, Marty-Ané C, Kovacsik H (2016). Normal postoperative appearances of lung cancer. Diagn Interv Imaging.

[7] Lim KA, Kim SH, Huh J, Kang IS, Lee HJ, Jun TG, Park PW (2005). Somatostatin for postoperative chylothorax after surgery for children with congenital heart disease. J Korean Med Sci.

